# Multidimensional Assessment of Interoceptive Abilities, Emotion Processing and the Role of Early Life Stress in Inflammatory Bowel Diseases

**DOI:** 10.3389/fpsyt.2021.680878

**Published:** 2021-06-24

**Authors:** Konstantina Atanasova, Tobias Lotter, Wolfgang Reindl, Stefanie Lis

**Affiliations:** ^1^Institute of Psychiatric and Psychosomatic Psychotherapy, Central Institute of Mental Health, Medical Faculty Mannheim, Heidelberg University, Mannheim, Germany; ^2^Department of Medicine II, Medical Faculty Mannheim, Heidelberg University, Mannheim, Germany; ^3^Department of Psychosomatic Medicine, Central Institute for Mental Health Mannheim, Medical Faculty Mannheim, Heidelberg University, Mannheim, Germany

**Keywords:** inflammatory bowel diseases, interoception, interoceptive sensibility, emotion processing, emotional awareness, early life stress, childhood trauma, heartbeat tracking

## Abstract

Perception of internal bodily sensations includes three dissociable processes: interoceptive accuracy, interoceptive sensibility, and interoceptive awareness. Interoceptive abilities play a crucial role in emotion processing and impairments of these processes have been reported in several psychiatric disorders. Studies investigating interoceptive abilities and their role in emotional experience in individuals with somatic disorders such as inflammatory bowel diseases (IBD) are sparse. Recent findings suggested an association between adverse childhood experiences (ACE) and the development of gastrointestinal disorders. The aim of the current study was to investigate the associations between the different dimensions of interoception and emotional processing in IBD while taking ACE into account. We recruited IBD patients in clinical remission (*n* = 35) and 35 healthy control participants (HC) matched for age, education and IQ. Interoception was measured as a three-dimensional construct. Interoceptive accuracy was assessed with the heartbeat tracking task and interoceptive sensibility with a self-report measure (Multidimensional Assessment of Interoceptive Awareness questionnaire). Emotional processing was measured using an experimental task, where participants were asked to rate the subjectively perceived valence and arousal when presented with positive, neutral and negative visual stimuli. IBD patients significantly differed in two interoceptive sensibility domains, Emotional awareness and Not-distracting. Patients reported greater awareness of the connection between bodily sensations and emotional states, while showing a stronger tendency to use distraction from unpleasant sensations compared with HC. Higher emotional awareness was linked to higher perceived intensity and arousal of negative stimuli. The strength of this relation was dependent on the severity of ACE, with severer traumatization being associated with a stronger association between emotional awareness and perceived valence and arousal. Our findings suggest that it is the subjective component of interoception, especially the one assessing interoceptive abilities within the scope of emotional experience, which affects emotional processing in IBD. This is the first study providing evidence that IBD patients did not differ in their perception of visceral signals *per se* but only in the subjective ability to attribute certain physical sensations to physiological manifestations of emotions. Our findings support the hypothesis that ACE affect the association between interoception and emotional processing.

## Introduction

Inflammatory bowel diseases (IBD) are immune mediated chronic diseases with a prevalence of 6.8 million cases globally. The incidence of IBD is continuously rising not only in industrialized countries of the northern hemisphere but also in developing countries (1). IBD include primarily Crohn's disease and Ulcerative colitis, both characterized by various physical and psychological symptoms such as abdominal pain, diarrhea, unwanted weight loss, depression, and anxiety (2). The etiology of IBD is multifactorial including genetic, immune, and environmental factors. While the impact of the environment on the development of IBD is substantial, the role of specific factors is still poorly defined, one candidate being early life stress (3–5). Newer studies in IBD have suggested alterations in the brain-body communication, directly influencing the sensory and immune functions of the gastrointestinal tract (6–8). In line, gastrointestinal pathologies have been recently linked to altered interoceptive processes (9). Interoception is closely linked to emotional processing (10, 11) and might influence the severity of IBD symptoms and disease course by increasing the level of stress through an increased sensitivity toward negative emotional stimuli. So far, studies on changes in interoception and their relation to emotion processing in IBD are sparse (12). The present study aims to contribute to the better understanding of these domains of functioning in IBD by investigating interoception as a multidimensional concept together with changes in emotion processing while taking the influence of early life stress into account.

Interoception refers to the sense through which bodily changes are signaled and perceived (13). It is conceptualized as a three-dimensional construct, comprising interoceptive accuracy (IACC), interoceptive sensibility (IS), and interoceptive awareness (IAw) (14). Interoceptive accuracy refers to the correspondence between the actual and the perceived bodily signals. In contrast, IS is defined as the subjective belief to be internally focused and represents the extent to which individuals feel engaged by interoceptive signals (14). IS includes not only individual's general awareness of bodily sensations but also beneficial and maladaptive forms of interoceptive engagement (15). Finally, interoceptive awareness represents the correspondence between interoceptive accuracy measured *via* behavioral tasks (e. g heartbeat tracking task) and subjectively reported general IS (16). It should be mentioned that the metacognitive component of interoceptive awareness should be measured only using interoceptive accuracy scores and respondent's ratings of the confidence in the accuracy of their performance in the applied task [for discussion see (107)]. In the present manuscript we retain the use of the term interoceptive awareness to refer to the correspondence between interoceptive accuracy scores and IS as measured *via* self-report measures. Interoceptive impairments have been linked to specific physical and mental health conditions, emphasizing the importance of precise body signals perception for the physical and mental well-being (17). Although impairments in interoception may contribute to the pathophysiology of gastrointestinal disorders, there is, to our knowledge, no study that investigated interoceptive processing in IBD as a multidimensional concept. Enhanced visceral perception sensitivity (“visceral hypersensitivity”) refers to the altered processing of visceral stimuli in gastrointestinal disorders (18). Current findings support the view that it results from disturbances along the brain-gut axis (19) and is related to functional alterations in brain regions involved in visceral afferent processing, emotional arousal and pain perception (20, 21). Visceral sensitivity in irritable bowel syndrome patients have been characterized by a biased appraisal of perceived bodily signals and enhanced selective attention to gastrointestinal sensations (20–23). Even though so far there is—to our best knowledge—no study investigating the link between visceral hypersensitivity and interoceptive processing, we suggest that IBD patients might exhibit stronger and more selective attention toward their bodily signals in order to recognize early signs of worsening disease activity and upcoming relapse, which might result in superior interoceptive abilities. To date, there are only few studies, which investigated interoceptive abilities in populations with gastrointestinal disorders. Findings suggest no alterations in the behavioral performance in interoceptive accuracy tasks, but only in self-reported IS in irritable bowel syndrome patients (24). In contrast, a study by Fournier et al. (25) found no differences in the interoceptive abilities of IBD patients measured with the Toronto Alexithymia Scale (26). Although it can be assumed that chronic exposure to visceral pain in IBD might be associated with superior abilities to detect bodily signals, experimental studies examining this association using both behavioral and self-report measures are missing. In IBD patients, the perception of physiological stimuli is critical for the regulation of psychological mood, which in turn may affect the appraisal of gastrointestinal symptoms and also the disease course.

Besides their role in symptoms perception, interoceptive abilities are of particular importance for many higher order cognitive processes such as learning, decision making, and emotion processing (27). The perception of physiological changes in the body is posited as a core component of emotional experience (13, 28). Based on neurobiological findings, Craig (29) postulated that interoception reflects the physiological condition of the entire body, including also the perception of the response of the body to different affective stimuli and its impact on emotional experience (29). Deficits in interoceptive abilities are associated with difficulties in identifying emotions, reduced emotional reactivity and inferior ability to downregulate negative emotions (30). In contrast, superior interoceptive abilities are linked to stronger associations between individual's body response to emotional stimuli and subjective ratings of perceived arousal (30, 31). Thus, altered processing of visceral signals, as observed in gastrointestinal disorders, may contribute to changes in patients' emotional functioning. A study by Vianna et al. (12) suggested the relevance of this link in IBD: the association between stronger visceral responses and higher subjective arousal to emotional stimuli was significantly increased in acute Crohn's disease patients compared to healthy controls (HC), particularly for negative stimuli (12). Additionally, findings from neuroimaging studies have indicated decreased sensitivity to positive emotional content in patients with Ulcerative colitis, suggesting the close connection between brain regions involved in the processing of interoceptive as well as emotional stimuli (32). Impairments in emotional functioning may have crucial negative impact on patients' well-being (e.g., depression, anxiety). Although it is conceivable that alterations in interoceptive abilities may partially explain impairments in emotion processing in IBD, studies investigating this link are sparse (25, 33).

One environmental factor that has been linked to an increased risk for mental and somatic disorders is a history of adverse childhood experiences (ACE). ACE, including emotional and physical abuse as well as neglect, have permanent consequences for the mental and somatic health in adulthood (34, 35). Individuals who report experiences of early life stress are more prone to developing chronic inflammation, cardiovascular disease and affective disorders compared to non-traumatized controls (36). Some authors emphasized a potential role of ACE in gastrointestinal disorders (4, 37). A cohort study revealed that the prevalence of at least one type of childhood adversity in IBD patients is above 70% with most of the patients reporting death of a family member during childhood and 12–13% reporting sexual or physical abuse (37). One biological mechanism, which may explain the negative impact of ACE on physical health in later life is the dysregulation of the hypothalamic-pituitary-adrenal (HPA) axis and alterations of the stress-response in individuals exposed to early life stress. Impaired physiological stress response affects the brain-body signaling, which in turn might lead to altered perception of bodily signals (38, 39). Beyond its negative impact on body signals perception, ACE have been also linked to impaired emotion regulation and emotional processing (40, 41). Thus, early life stress might lead to dysregulation of the brain-body axis and altered interoception, leading to impairments of emotional processing (42). However, the role of childhood adversity as vulnerability factor for altered perception of bodily signals and impaired emotional functioning in IBD is still unclear (43). Besides higher rates of ACE, newer findings suggest that one third of the IBD patients meet the criteria for clinically important symptoms of a post-traumatic stress disorder (PTSD), and patients attribute these to their disease (44). Thus, histories of childhood traumatization, as well as chronic stress associated with the disease experience itself constitute stressors, which may lead to changes in arousal, physiological reactivity and emotional experience.

The aim of the current study was to investigate alterations of interoceptive abilities and emotion processing in IBD compared to healthy participants. We proposed that the chronic exposure to gastrointestinal symptoms may lead to enhanced attention toward bodily signals, resulting in superior interoceptive abilities in IBD. The perception of physiological changes in the body is a core component of emotional experience and greater interoceptive accuracy is linked to more intense emotional experience and greater perceived arousal. Therefore, changes in visceral signals perception might explain altered emotional processing among IBD patients. To the best of our knowledge, this is the first study investigating interoception as a multidimensional construct and its effects on emotion processing in IBD by combining behavioral and self-report measures. Measures of interoception included interoceptive accuracy using the heartbeat tracking task (HBT) (45), IS assessed *via* self-reports (46) and interoceptive awareness as the correspondence between interoceptive accuracy and IS. We focused on IBD patients in clinical remission to avoid a potentially confounding effect of active disease symptoms on the interoceptive measures. For the same reason we chose to assess interoceptive accuracy using the HBT. A high correlation between measures of cardiac and gastric interoception points to the presence of a general sensitivity for interoceptive cues across different sensory modalities (47, 48). In line, we used the HBT as a substitute for measures of gastric interoceptive accuracy, since it is less susceptible to confounding effects of abdominal pain in this patients' group. HBT is a well-established method to measure interoceptive accuracy that is typically performed under conditions of physical rest (45). However, when studying the relationship between interoception and emotional experience, an assessment of interoceptive accuracy under physical rest might fail to measure important characteristics of the cardiovascular system such as its dynamic responses to arousing stimuli (49). To overcome this shortcoming, we implemented a short physical exercise with the purpose of inducing a physiological reaction in our participants. According to previous findings by Schaan et al. (38), increased heartrate resulting from a physical activation should lead to improvement in participants' interoceptive accuracy (50).

According to this rationale, we hypothesized that IBD patients will (I.) demonstrate superior interoceptive accuracy and IS, as well as greater congruency between these constructs and (II.) report higher arousal and emotional intensity when evaluating negative stimuli compared with a healthy control group. We hypothesized that (III.) greater interoceptive accuracy will be associated with higher arousal and emotional intensity ratings during emotional processing. Moreover, we expected that ACE will modulate alterations in interoception and emotional processing and strengthen the association between interoceptive accuracy and emotional processing. (IV.) We expected the performance of a short physical exercise to result in improved interoceptive accuracy. Therefore, we explored whether group differences in interoceptive accuracy increased after performing a physical challenge. Finally, we investigated whether heart rate and heart rate variability during the interoception and emotion processing tasks differed between IBD patients and healthy individuals and expected (V.) a decreased heart rate variability (HRV) in the IBD group compared with healthy participants as repeatedly shown in the literature before (51).

## Materials and Methods

### Participants

The study was approved by the Ethics Committee of the Medical Faculty Mannheim at Heidelberg University and all participants gave their written informed consent before participating in the study. The hypotheses, sample size, methods, exclusion criteria and planned analyses were preregistered before data collection and can be accessed at: https://aspredicted.org/blind.php?x=dj6fz9. All aspects of the study were carried out in accordance with the pre-registered protocol unless otherwise stated.

In total, 70 individuals in age 18-65 years participated in the study. Of these, 35 met the diagnostic criteria for IBD and 35 were HC. Both groups were matched for age, IQ [Multiple-Choice Word Test-B (MWT-B); (52)] and education. For age, we accepted a difference of ± 5 years in pairwise matches between patient's and HC's age. We aimed to achieve two samples comparable on a group level. Since equal education level was of particular importance for our matching procedure, we matched each healthy participant with the same education as the one reported by the IBD patient. Therefore, the final sample included more female participants in the HC group than in the IBD group. For further details, see Table 1. We excluded one participant of the IBD group from further analyses, since she/he reported no perceived heartbeats during the HBT. Thus, the final sample consisted of 34 IBD patients and 35 HC. According to our preregistered analyses a sample size of 120 participants was planned. However, due to the Covid-19 outbreak and the pandemic-related restrictions the number of participants was reduced.

**Table 1:** Sample characteristics including demographic data, affective state prior measurement and psychological well-being.

	**IBD (M±SD)**		**HC (M±SD)**		**Test-statistics**	***P*-value**
**Demographics**						
Age	41.32	±14.36	37.06	±11.96	1.34^a^	0.184
Sex (female/male)	18/16		20/15		0.12^b^	0.726
BMI	25.09	±3.32	24.59	±4.52	0.52^a^	0.607
Years of education	12.44	±3.11	12.97	±2.62	−0.77^a^	0.445
MWT-B	29.79	±4.05	30.51	±3.71	−0.77^a^	0.443
**Affective state**						
STAI Anxiety (state)	34.35	±5.82	32.23	±8.39	0.99^a^	0.327
SAM-Valence	3.64	±0.99	3.81	±0.85	−0.69^a^	0.492
SAM-Arousal	2.51	±0.98	2.03	±0.93	2.06^a^	0.043^*^
SAM-Dominance	3.48	±0.62	3.70	±0.70	−1.04^a^	0.302
**Psychological distress**						
GSI	28.90	±9.38	23.50	±5.94	2.71^a^	0.009^**^
Somatization	9.63	±3.11	6.94	±1.56	4.29^a^	< 0.001^***^
Depression	9.47	±3.71	8.00	±3.24	1.69^a^	0.096
Anxiety	9.80	±3.84	8.56	±2.60	1.53^a^	0.131
**Visceral sensitivity**						
VSI	28.87	±14.95	–		–	–

*IBD, inflammatory bowel diseases group; HC, healthy controls group; BMI, body-mass index; MWT-B, multiple choice word test-B; STAI, state-trait anxiety inventory; SAM, self-assessment manikin; GSI, general symptom index; VSI, visceral sensitivity index.*

* *p < 0.05,*

** *p < 0.01,*

*** *p < 0.001,*

a *T-value,*

b *Chi^2^ statistic*.

IBD patients were recruited from the IBD outpatient unit at the University Medical Center Mannheim. Overall, 23 patients with Crohn's disease and 11 with ulcerative colitis in clinical remission were included in the IBD group. Crohn's disease patients exhibited mean Harvey-Bradshaw Index score of 1.43 ± 1.56 and UC patients mean Partial Mayo Score of 1.73 ± 1.49. The average age for disease onset was 25.65 ± 10.83 years. The average disease duration was 15.67 ± 13.55 years. Overall, 10 IBD patients reported a history of Extraintestinal manifestations (Enteropathic arthritis *n* = 6, Dermatitis *n* = 4). Nineteen patients did not report histories of previous surgeries, while the remaining IBD patients underwent surgeries including ileal resection, ostomy, fistula removal and abscess drainage. Diagnostic procedures and gastroenterological examinations were carried out on all patients by fully trained physicians specialized in the care of patients with IBD. Exclusion criteria were biological signs of disease activity (fecal calprotectin level in mg/L > 200), current use of corticosteroids, use of psychotropic medications other than SSRIs or SNRIs, and current or prior history of neurological or mental disorders. Thirty-three patients reported a current treatment with biologics and one using Mesalazine. Lastly, ten IBD patients reported having histories of further somatic diseases (pulmonary embolism *n* = 2, pancreatitis *n* = 2, diabetes *n* = 1, chronic cystitis *n* = 1, thrombocythemia *n* = 1, asthma bronchiale *n* = 1, and hypothyroidism *n* = 2). Exclusion criteria for the HC group were chronic medical conditions, chronic medication intake, use of psychotropic medication, and current or prior history of neurological or psychiatric disease and general gastrointestinal complaints (e.g., abdominal pain, diarrhea) during the last 4 weeks prior to testing. For further details on sample characteristics, see Table 1.

To characterize the sample, we assessed psychological distress with the Brief Symptom Inventory (BSI-18) (53). BSI-18 is a is an 18 Items self-report instrument that measures somatization, depression, and anxiety in three subscales with 6 items each and a global symptom severity score [Global Severity Index (GSI)]. BSI-18 is a reliable instrument for measuring psychological distress and comorbidities in patients with mental and somatic disorders (54, 55). The scores for the GSI range from 0 to 72 points and from 0 to 24 points for each subscale with higher scores indicating higher symptom severity. GSI total scores showed acceptable reliability in the present study, however the reliability of the subscale scores suggests that they have to be interpreted with care (IBD: Cronbach's α = 0.88, 0.61, 0.76, 0.83; HC: α = 0.85, 0.66, 0.87, 0.66).

Prior to testing, participants evaluated their current affective state with the state version of the State-Trait Anxiety Inventory [STAI; (56)]. It is a 20 items scale, which measures subjective feelings of apprehension, tension, nervousness, worry, and activation/arousal of the autonomic nervous system on a 4-point Likert scale (1 “not at all” to 4 “very much so”). Items scores are added to obtain scale total score (range from 20 to 80) with higher scores indicating greater anxiety. Cronbach's α in the current study was α = 0.79 in the IBD sample and 0.91 in the HC group. Additionally, arousal, valence, and dominance levels were assessed *via* the Self-Assessment Manikin [SAM; (57)]. SAM is a non-verbal pictorial assessment technique that measures valence/pleasure, perceived arousal, and perceptions of dominance on a 9-point Likert scale. Higher scores indicate greater positive valence, higher arousal and higher perceived dominance.

Finally, visceral sensitivity was measured with the Visceral Sensitivity Index [VSI; (58)]. The 15-item scale assesses gastrointestinal symptom-specific anxiety, comprising Worry, Fear, Vigilance, Sensitivity, and Avoidance as gastrointestinal-related cognitions and behaviors. Items are scored on a reversed 6-point scale ranging from 0 “strongly agree” to 5 “strongly disagree.” VSI has been developed specifically for patients with functional gastrointestinal disorders. Since the VSI is not a suitable measure for healthy individuals,' we utilized this self-report measure in the IBD group only. The overall VSI score ranges from 0 to 75 points with higher scores indicating more severe gastrointestinal-specific anxiety, as well as lower tolerance to visceral pain. Reliability in the current sample was Cronbach's α = 0.89.

### Procedure

**Figure 1:**
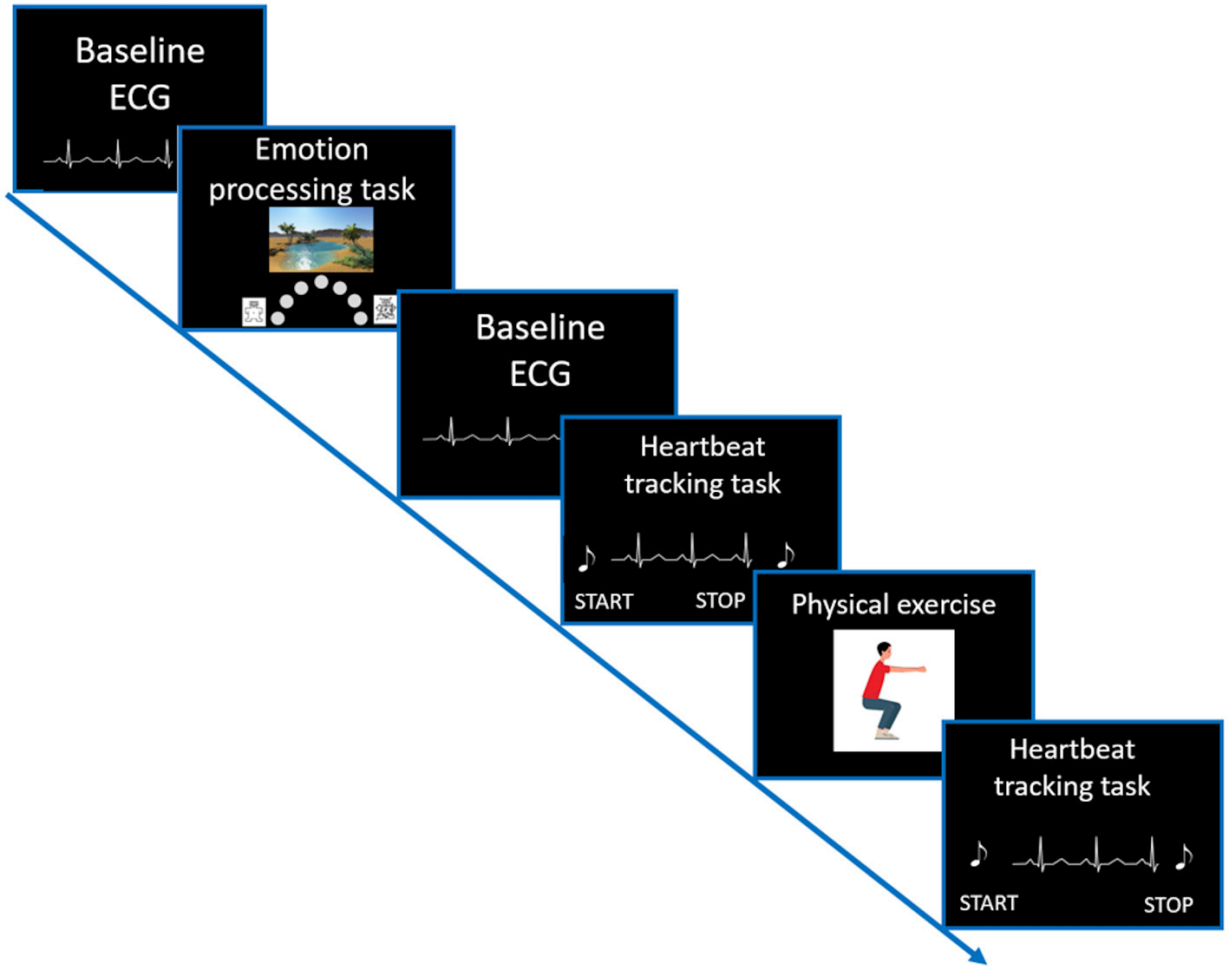
Visualization of the study setup. After completing all questionnaires, a resting ECG (duration of 2 min) was recorded. Afterwards, participants performed an emotion processing task. Again, before starting the Heartbeat tracking task, a resting ECG recording was conducted. For more details on the trial structure of the Heartbeat tracking task please see Figure 2.

Participants evaluated their general interoceptive sensibility by a self-report questionnaire. Afterwards, the ECG assessment was started, and participants were asked to perform three experimental tasks, including an emotional processing task and the heartbeat tracking task followed by a time estimation task (see Figure 1).

### Questionnaires

#### Interoceptive Sensibility: MAIA

We assessed IS using the Multidimensional Assessment of Interoceptive Awareness [MAIA; (46, 59)]. The 32 items self-report questionnaire measures eight different facets of interoceptive sensibility on a 6-point Likert-scale (0 = “never”− 5 = “always”) with higher scores indicate greater sensitivity to signals from the body. MAIA provides a multidimensional profile of body awareness including the following eight subscales: *Noticing* (being aware of body sensations), *Not-Distracting* (being inclined to not distract or ignore painful or uncomfortable sensations), *Not-Worrying* (inclination to not be emotionally distressed by uncomfortable sensations), *Attention Regulation* (paying attention to and controlling attention on body sensations), *Emotional Awareness* (being aware of the connection between emotions and body sensations), *Self-Regulation* (regulating distress through paying attention to body sensations), *Body Listening* (purposefully listening for insight from the body) and *Trusting* (experiencing trust with and safety in the body) (46). MAIA revealed good internal consistency in the current experiment with Cronbach's α = 0.91 in the IBD group and 0.94 in the HC group. A key advantage of MAIA is the possibility to assess various aspects of IS, including subscales distinguishing between beneficial and dysfunctional forms of IS. While “*Noticing*” assesses the awareness of bodily sensations in general, “*Attention Regulation*” and “*Not-Distracting*” measure individual differences in how engaged a person is regarding interoceptive cues (59). Moreover, previous findings indicated that general IS (“*Noticing*” subscale) did not benefit from body-focused interventions, aiming at improving interoceptive abilities, while the ability to sustain and control attention to bodily sensations (“*Attention Regulation*” subscale) showed a significant improvement (15). The postulated association between bodily sensations and emotional experience is assessed by the subscale “*Emotional Awareness*.” Since discrete emotions are linked to physiological reactions, individuals might differ in terms of their ability to recognize these mind-body associations, which might lead to difficulties in recognizing their emotional state.

#### Childhood Traumatization and PTSD Symptoms Severity

We assessed traumatization during childhood and adolescence as well as traumatic experiences during lifetime using a battery of psychometric measures. Severity of ACEs were measured with the German version of the Childhood Trauma Questionnaire [CTQ; (60)]. This 28-item questionnaire (five-point Likert scale ranging =1 “not at all” −5 = “very often”) has been shown to have good psychometric properties in previous research (60). The CTQ consists of five subscales including emotional abuse (Cronbach's α in this study 0.79), physical abuse (α = 0.73), sexual abuse (α = 0.88), emotional neglect (α = 0.84), and physical neglect (α = 0.45) and a total score (Cronbach's α in IBD: 0.86; HC: 0.73). Each subscale is represented by five questions with a score from 5 to 25. Scores fall into four severity categories: none to low, low to moderate, moderate to severe and severe to extreme traumata exposure for each subscale (see Table 2).

**Table 2:** Sample characteristics including childhood traumatization, traumatization in later life, and PTSD symptoms severity.

	**IBD (M** **±** **SD)**		**HC (M** **±** **SD)**		**Test-statistics**	***P*-value**
**TRAUMA HISTORY**						
**Childhood trauma**						
CTQ total	35.73	±10.77	36.06	±7.00	−0.15^a^	0.881
None to minimal ACE	*n* = 21		*n* = 21		0.10^b^	0.758
Low to moderate ACE	*n* = 10		*n* = 13		0.36^b^	0.551
Moderate to Severe ACE	*n* = 2		*n* = 1		0.41^b^	0.520
CTQ—EA	8.18	±3.73	8.51	±2.96	−0.41^a^	0.684
CTQ—PA	6.27	±2.53	5.80	±1.60	0.92^a^	0.362
CTQ—SA	5.55	±2.00	5.14	±0.55	1.12^a^	0.272
CTQ—EN	8.91	±3.79	9.63	±3.29	−0.84^a^	0.405
CTQ—N	6.81	±2.34	6.97	±1.71	−0.31^a^	0.758
CECA (loss/death of a parent)	*n* = 10		*n* = 9		0.26^b^	0.608
**Lifetime trauma**						
LEC-5	1.52	±1.56	1.11	±1.30	1.15^a^	0.254
**PTSD symptoms**						
PCL-5 total	15.47	±12.37	5.54	±5.94	4.13^a^	<0.001^***^
Cluster B	4.25	±3.69	1.43	±1.95	3.86^a^	<0.001^***^
Cluster C	1.81	±2.13	0.74	±1.20	2.50^a^	<0.013^*^
Cluster D	4.16	±3.55	1.86	±2.79	2.96^a^	0.004^**^
Cluster E	4.84	±4.52	1.43	±1.70	4.02^a^	<0.001^***^

*IBD, inflammatory bowel diseases group; HC, healthy control group; CTQ, childhood trauma questionnaire; EA, emotional abuse; PA, physical abuse; SA, sexual abuse; EN, emotional neglect; PN, physical neglect; ACE, adverse childhood experiences; CECA, childhood experience of care and abuse; LEC-5, life events checklist; PTSD, post-traumatic stress disorder; PCL-5, post-traumatic stress disorder checklist for DSM-V; Cluster B = intrusion symptoms, Cluster C = avoidance, Cluster D = negative alterations in cognitions and mood, Cluster E = alterations in arousal and reactivity,*

* *p < 0.05,*

** *p < 0.01,*

*** *p < 0.001,*

a *T-value,*

b *Chi^2^ statistic*.

Furthermore, to assess additional types of early life stress such as parental loss, continuous separation from one or both parents and foster care experiences we used selected items (1a, 1b, 2a, 2b) of the Childhood Experience of Care and Abuse Questionnaire [CECA.Q, (61); German version: (62)].

Additionally, all participants were screened for potentially traumatic life events using the Life events Checklist for DSM-5 [LEC-5; (63)]. It is a self-report measure, which assesses exposure to 16 events known to potentially result in symptoms of posttraumatic stress disorder (PTSD) or distress. LEC-5 distinguishes not only between event types (e.g., physical or sexual assault, accident) but also between the ways a person was exposed to the stressor (e.g., direct exposure or witnessing the traumatic event). For each participant, the number of personally experienced traumatic events (direct exposure) were summed up, resulting in one sum score.

Severity of PTSD symptoms was evaluated with the PTSD Checklist for DSM-5 [PCL-5; (63)]. It is a 20-item self-report measure that assesses the presence and severity of symptoms after experiencing a traumatic event. Items correspond with DSM-5 criteria for PTSD including intrusion symptoms, avoidance, negative alterations in cognitions and mood and alterations in arousal and reactivity during the last 4 weeks. The rating scale ranges from 0 “not at all” to 4 “extremely” for each symptom. A total symptom severity score is obtained, with higher scores indicating higher PTSD symptoms severity. PCL-5 revealed high reliability in the current study with Cronbach's α = 0.91 in IBD and.87 in HC. See Table 2.

### Experimental Tasks

#### Interoceptive Accuracy: HBT

**Figure 2:**
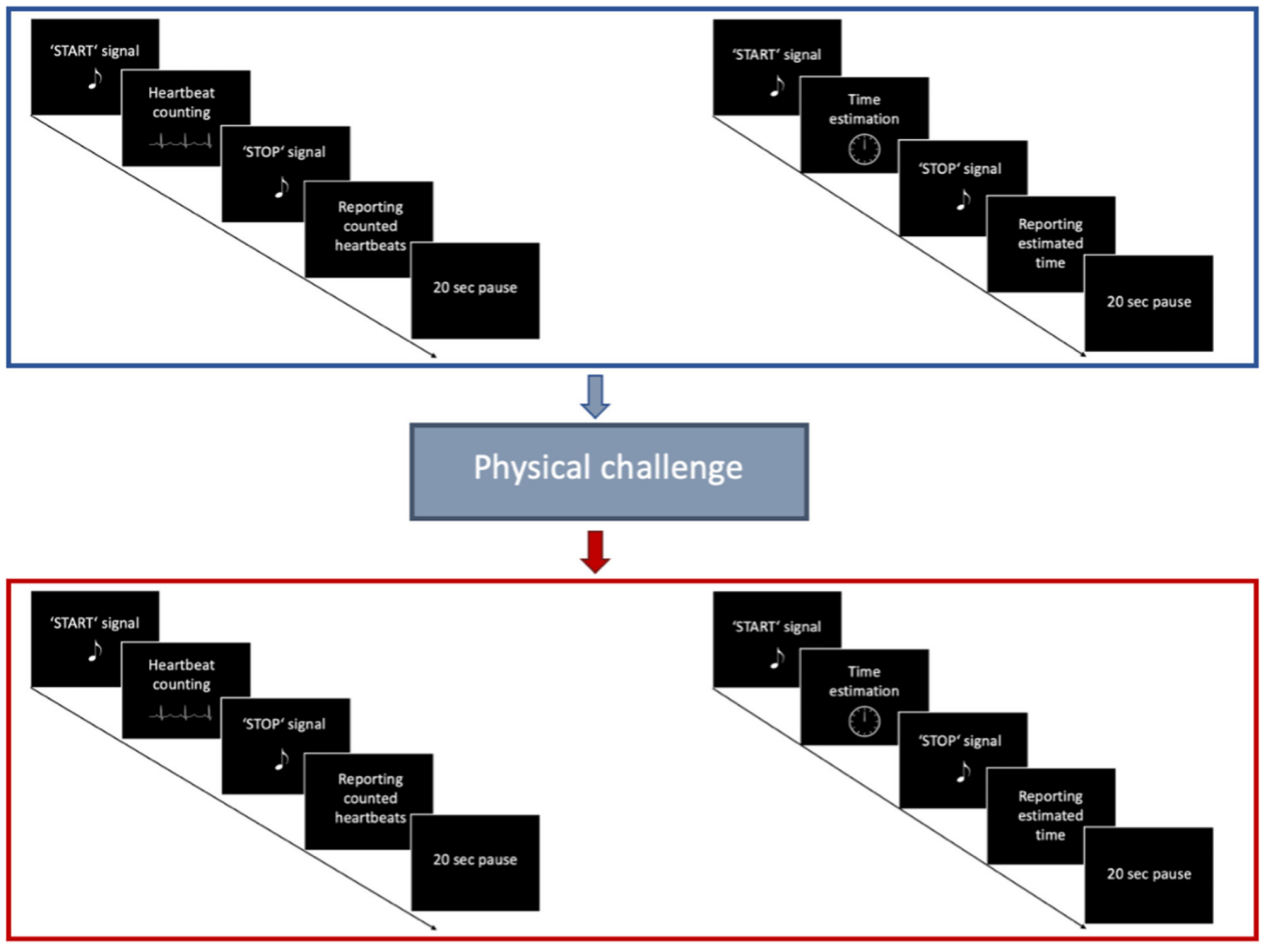
Trial structure of the Heartbeat tracking task. First, participants performed the HBT for five different time intervals in a pseudorandomized order. Afterwards, they performed a Time estimation task for the same time intervals. After a short physical challenge of 10 squats was implemented, participants performed once again the HBT, followed by the Time estimation task.

Interoceptive accuracy was measured with a standard heartbeat tracking task [HBT, (45)]. Participants were seated upright in a quiet room with their eyes closed. They were asked to silently count the number of heartbeats occurring during five discrete time intervals of 25, 35, 45, 55, and 100 s. Participants were explicitly told not to estimate their heartbeats, but to count only those heartbeats they actually perceived. Intervals varied in their length and were presented in a randomized order counterbalanced across both groups. Before starting with the experimental task, a baseline measurement of cardiac activity was performed (Figure 1). Each HBT interval started after a resting period of 20 s. While counting their heartbeats, participants were not allowed to use a manual pulse. To explore the effects of dynamic alterations in the cardiovascular homeostasis on interoceptive accuracy, all participants were asked to perform a short physical exercise comprising 10 squats (Figure 2). However, some of the IBD patients reported pain in the knees and refrained from performing this exercise. Therefore, data for the second run of the task was available only for a subsample of participants (24 IBD patients and 24 HC). To compute interoceptive accuracy scores, the ratio of the number of reported heart beats and number of R-waves in the ECG was calculated for each of the time intervals of the HBT and averaged across the five intervals [1/5 ∑ (1-∣actual heartbeats-counted heartbeats∣/actual heartbeats)]. In the present sample, interoceptive accuracy scores ranged from 0 to 1 (no over-reporters observed) with higher scores indicating greater interoceptive accuracy.

#### Time Estimation Task

Subject's performance on the HBT might be confounded by individuals' abilities to estimate time, and their knowledge about their own heart rate (64, 65). Therefore, we extended the experimental task by an additional time estimation task (Figure 2) (10). Participants were asked to estimate the elapsed time during five discrete time intervals of 25, 35, 45, 55 and 100 s. Time intervals were presented in pseudorandomized order, counterbalanced across the experimental groups. The time estimation task was performed after the HBT prior and following the physical challenge. Similar to the computation of interoceptive accuracy scores, time estimation (TE) accuracy was calculated using the following formula [1/5 ∑ (1–∣actual elapsed time—estimated elapsed time∣/actual elapsed time)] (10).

#### Emotional Processing Task

Emotional processing was measured by ratings of subjective intensity of arousal and valence when being presented with emotional stimuli. The task involved 90 pictures of three categories (30 positive, 30 negative, and 30 neutral pictures) from the OASIS data base [(66) www.benedekkurdi.com/#oasis], presented in a pseudorandomized order. Based on existing norms, “negative” stimuli were selected based on high arousal (M = 4.12, SD = 0.45) and low valence ratings (2.58 ± 0.35), “positive” pictures had high arousal (4.15 ± 0.48) and high valence ratings (6.04 ± 0.23), and “neutral” stimuli had low arousal (2.36 ± 0.53) and moderate valence rating (4.08 ± 0.10). Emotional stimuli were presented on a computer screen and participants were asked to rate their subjectively experienced level of arousal and valence for each stimulus. Arousal and valence ratings were made in two separate blocks, each one consisting of 90 pictures, resulting in overall 180 trials. All ratings were performed on an 8-point scale (valence: 1 = “very unpleasant” − 8 = “very pleasant”; arousal: 1 = “not aroused at all” − 8 = “highly aroused”) by moving a mouse cursor to one of the eight target buttons. Trials were self-paced, with participants signaling the start of the trial by moving the cursor to a start button that was placed in equal distance from the semicircular arranged target buttons.

#### ECG Recording and Preprocessing

Electrocardiogram (ECG; Einthoven II) was obtained by attaching two one-way hydrogel electrodes (Kendall™ Covidien, Germany) under the right and the left clavicle, respectively, and a third electrode was placed on the left side under the lowest rib. Signals were recorded with a sample rate of 1,024 Hz using a Varioport system (Becker, meditec). Participants were instructed to avoid body movements and to breathe in their natural manner. ECG was recorded during each experimental task as well as during two resting periods with a duration of 2 min each (Figure 1).

R-waves of the QRS-complex were determined by the Pan-Tompkins algorithm (67, 68) using custom written scripts in Matlab 2020a (The Mathworks, Inc.). Accuracy of the automatic detection of R-waves was subsequently controlled by visual inspection to identify ectopic heart beats and time intervals confounded by artifacts. Following this preprocessing procedure, the number of heart beats for each of the time intervals of the HBT was determined and stored for further calculation of the interoceptive accuracy scores. Additionally, heart rate (HR) and root mean square of the successive differences (RMSSD) were calculated separately for the HBT (averaged across all time intervals), the emotional processing task, as well as the resting ECGs recorded prior to the experimental tasks (69). We calculated RMSSD of the normal-to-normal beat intervals (NN), while excluding each interval with an ectopic beat as well as the preceding and following RR interval, since these might be also distorted by the occurrence of ectopic beats (70). In general, RMSSD is considered to be a robust and statistically reliable HRV marker (71). It represents short-term HRV (72) and is used as a marker for vagally mediated HRV.

### Statistical Analyses

Statistical analyses were carried out with SPSS v.25.0 (IBM Corp., USA). For all analyses statistical significance was set to *p* < 0.05.

#### Interoceptive Accuracy and Accuracy of Time Estimation

Interoceptive accuracy und TE accuracy scores were compared between experimental groups with independent samples *t*-tests. Additionally, we analyzed the effects of the physical challenge in a subsample of participants, for interoceptive accuracy and time estimation accuracy in two separate 2 ×2 mixed ANOVAs with the independent factor “group” and the repeated measurement factor “time” (pre/post physical challenge).

#### Interoceptive Sensibility

To test group differences in IS, mean scores of all MAIA subscales were analyzed with multiple independent samples *t*-tests.

#### Interoceptive Awareness

Interoceptive awareness is defined as the correspondence between one's interoceptive accuracy, measured with an objective behavioral task, and IS that is the subjective evaluation of interoceptive abilities assessed *via* self-report measures. Previous studies investigating the link between behavioral measures of interoception (e.g., HBT) and MAIA subscales revealed associations only with the subscale “*Attention Regulation*” (73). Thus, it has been suggested that interoceptive accuracy measured by the HBT relates to more specific aspects of IS and not so much to general IS as assessed by the “*Noticing*” subscale. Since the HBT requires individual's ability to control and maintain attention toward bodily sensations, we assessed interoceptive awareness by computing Pearson's correlation coefficients between interoceptive accuracy scores and IS_attention_ _regulation_ scores across all participants in each experimental group (14, 74). To investigate group differences in interoceptive awareness, correlation coefficients in the IBD and HC group were standardized and compared between groups (75).

#### Emotional Processing

Differences in emotion processing between IBD and HC were analyzed with two separate 2 ×3 mixed-effects ANOVAs for the two dependent variables “valence” and “arousal” with the between-subject factor “group” and within factor “stimulus valence” (positive/ neutral/negative). Degrees of freedom were corrected using Greenhouse-Geisser estimate of sphericity. *Post-hoc* comparisons were done by sub-designs of ANOVA design and/or pairwise comparisons.

#### The Link Between Interoceptive Accuracy and Emotional Processing

To investigate whether greater interoceptive accuracy was associated with higher levels of experienced valence and arousal during emotional experience, two linear regression analyses across all participants were computed. Valence and arousal ratings averaged separately for the three types of stimuli valence constituted the dependent variables (DV), while interoceptive accuracy scores were used as independent variable (IV). Beyond these preregistered analyses, we computed two additional linear regressions to test whether superior IS was associated with changes in perceived valence and arousal of emotional stimuli. Since we hypothesized that alterations in emotional processing linked to interoceptive processes will be more pronounced for negative stimuli, mean valence and mean arousal ratings only for negative stimuli were included in the analyses.

#### The Role of ACE in Interoception, Emotional Processing and Their Association in IBD

The effect of childhood traumatization on interoceptive accuracy and IS was investigated using two linear regression analyses with CTQ scores (IV) and interoceptive accuracy and IS scores (DV), respectively. Furthermore, to investigate whether severity of childhood traumatization does influence emotional processing, linear regression models for valence and arousal ratings were computed separately. Finally, the modulating effect of ACE on the relationship between interoceptive accuracy and emotional processing was investigated using separate moderation analyses with interoceptive accuracy scores (IV) and valence and arousal ratings (DV), respectively. Again, beyond the preregistered statistical analyses, we computed two additional moderation models with IS (IV) and valence and arousal ratings (DV).

#### Heart Rate and Heart Rate Variability

HR and RMSSD were compared between groups with independent *t*-tests. The effects of the physical activation on HR and HRV parameters were analyzed with a variance analytical design including the independent factor “group” and the repeated measurement factor “time” (pre/post challenge) in the subgroup of participants who performed the short physical exercise. Finally, differences between baseline cardiac activity and physiological activation during the emotional processing were analyzed with a variance analytical design including the independent factor “group” and the repeated measurement factor “time” (baseline/task).

To control for multiple testing, we report the corresponding *p*-values adjusted according to Benjamini and Hochberg (76) and indicated this by a subscript (*p*_*FDR*_).

## Results

### Interoceptive Accuracy and Time Estimation Accuracy

Comparisons between IBD and HC revealed no differences between both groups either for the interoceptive accuracy scores or the TE scores (IACC *t*_67_ = −0.432, *p* = 0.667, TE *t*_67_ = −0.047, *p* = 0.963, see Table 3).

**Table 3:** Group differences in interoceptive accuracy, interoceptive sensibility and interoceptive awareness.

	**IBD (M** **±** **SD)**		**HC (M** **±** **SD)**		**Test-statistics**	***P*-value**
**Interoceptive accuracy**						
IACC	0.65	±0.19	0.67	±0.21	−0.432^a^	0.667
TE accuracy	0.73	±0.14	0.73	±0.15	−0.047^a^	0.963
**Interoceptive sensibility**						
MAIA Noticing	2.61	±1.14	2.74	±1.03	−0.492^a^	0.625
MAIA Not distracting	2.20	±1.09	3.10	±0.83	−3.500^a^	0.004^**^
MAIA Not worrying	2.67	± 1.10	2.88	±0.79	−0.929^a^	0.356
MAIA Attention regulation	2.65	±0.89	2.41	±0.68	1.243^a^	0.218
MAIA Emotional awareness	3.47	±0.83	2.72	±0.92	3.504^a^	0.004^**^
MAIA Self-regulation	2.32	±1.03	1.95	±0.91	1.561^a^	0.123
MAIA Body listening	1.95	±1.16	1.76	±1.10	0.683^a^	0.497
MAIA Trusting	2.98	± 1.29	3.04	±1.05	−0.205^a^	0.839
**Interoceptive awareness**						
IAw	−0.317^b^		0.049^b^		−1.49^c^	0.069(^*^)

*IBD, inflammatory bowel diseases group; HC, healthy control group; IACC, interoceptive accuracy; MAIA, multidimensional assessment of interoceptive awareness; IAw, interoceptive awareness.*

* *p < 0.05,*

** *p < 0.01,*

a *T-value,*

b *r,*

c *Z-value*.

An analysis of the effect of the physical challenge in a subgroup of participants showed also no group differences for interoceptive accuracy either in general or depending on the challenge (main effect “group”: *F*_1, 46_ = 0.108, *p* = 0.744; “group” × “time”: *F*_1, 46_ = 0.777, *p* = 0.383). However, interoceptive accuracy after the physical challenge improved as non-significant trend (main effect “time”: interoceptive accuracy: *F*_1, 46_ = 2.864, *p* = 0.097). In contrast to interoceptive accuracy, HCs improved significantly in their TE performance after the physical challenge, whereas IBD patients did not show a significant difference (main effect “group”: *F*_1, 46_ = 1.860, *p* = 0.179; “group” × “time”: *F*_1, 46_ = 4.428, *p* = 0.041).

### Interoceptive Sensibility

IBD patients reported a stronger tendency to distract themselves from unpleasant sensations (IS_not-*distracting*_: *t*_66_ = −3.500, *p*_*FDR*_ = 0.004) and superior awareness of physical sensations associated with emotional states (IS_emotional awareness_: *t*_66_ = 3.504, *p*_*FDR*_ = 0.004). For further details on MAIA subscales see Table 3.

### Interoceptive Awareness

Interoceptive accuracy and IS_attention regulation_ scores showed a non-significant trend association in the IBD group but not in HC (all participants: *r*_68_ = −0.151, *p* = 0.218; IBD: *r*_33_ = −0.317, *p* = 0.072; HCs group: *r*_35_ = 0.049, *p* = 0.779). Interoceptive awareness differed between groups only as a non-significant trend (*Z* = −1.49, *p* = 0.069; Table 3).

### Emotional Processing Task

Valence ratings did not differ between both groups, either in general or dependent on the stimulus valence (main effect “group”: *F*_1, 64_ = 2.08, *p* = 0.154; “group” × “stimulus valence”: *F*_1, 84_ = 0.80, *p* = 0.407) (Table 4).

**Table 4:** Results of the analyses of variance for mean valence and arousal ratings with between-subject factor “group” (IBD/HC) and within-subjects factor “stimulus valence” (positive/neutral/negative).

**Emotional processing task**			
	***F***	***df*1/*df*2**	***P*-value**
**Valence ratings**			
Group	2.08	1/64	0.154
Stimulus valence	301.63	1/84	<0.001^***^
Group × stimulus valence	0.80	1/84	0.407
**Arousal ratings**			
Group	0.43	1/64	0.517
Stimulus valence	312.54	1/96	<0.001^***^
Group × stimulus valence	3.64	1/96	0.042^*^

*df, degrees of freedom;*

* *p < 0.05,*

** *p < 0.01*.

Arousal ratings differed between groups depending on the stimulus valence (“group” × “stimulus valence”: *F*_1, 96_ = 3.64, *p* = 0.042; main effect “group”: *F*_1, 64_ = 0.43, *p* = 0.517). *Post-hoc* analyses revealed a significant interaction effect “group” × “stimulus valence” (*F*_1, 64_ = 5.329, *p* = 0.024) with IBD patients exhibiting a greater difference in their perceived arousal between neutral and positive stimuli compared to HC (IBD: M_neutral_ = 5.10 ± 1.08, M_positive_ = 7.11 ± 0.54; HC: M_neutral_= 5.45 ± 1.28, M_positive_=6.79 ± 1.01).

### Interoception and Emotional Processing

**Figure 3:**
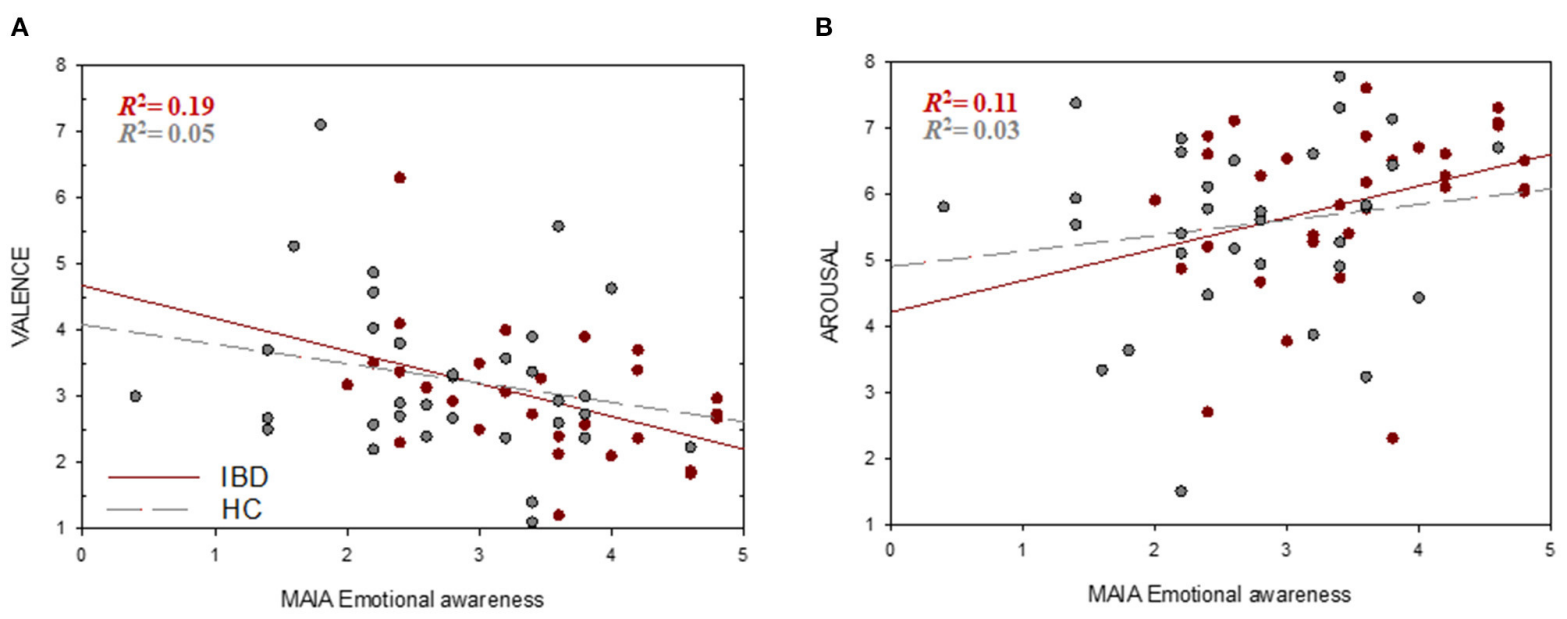
Associations between interoceptive sensibility and emotional processing. Higher IS_emotional awareness_ (MAIA) scores were associated with higher higher negative valence (1 = very unpleasant, 8 = very pleasant) **(A)** and arousal ratings (1 = low arousal, 8 = high arousal). **(B)** of negative stimuli.

Regression analyses revealed no significant associations between interoceptive accuracy and subjective valence or arousal ratings (all *p* > 0.05). Since only IS_emotional awareness_ and IS_not-*distracting*_ scores revealed significant group differences, only these subscales were used to investigate the link between IS and emotional processing. IS_emotional awareness_ was a significant predictor of perceived valence of negative stimuli in the whole sample (*b* = −0.390, *p*_FDR_ = 0.012) with participants reporting greater emotional awareness perceiving negative stimuli as more negative compared to those with lower emotional awareness. Further analyses revealed that this association was significant only in the IBD group (*b* = −0.493, *p*_FDR_ = 0.024) but not in the HC group (*b* = −0.293, *p*_FDR_ = 0.371) (Figure 3A). Similarly, IS_emotional awareness_ showed a significant association with perceived arousal of negative stimuli for all participants (*b* = 0.357, *p*_FDR_ = 0.034), with greater emotional awareness being associated with higher arousal. A non-significant trend for this association was found in the IBD group (*b* = 0.477, *p*_FDR_ = 0.069) but not in HC (*b* = 0.235, *p*_FDR_ = 0.371) (Figure 3B). IS_not-*distracting*_ was not significantly associated with emotional processing (all *p* > 0.05).

### Group Differences in Childhood Traumatization, Later Traumatic Experiences and PTSD Symptom Severity

There were no significant differences between both groups regarding the severity of ACEs measured with the CTQ. An equal number of IBD patients and HCs reported separation or death of a parent during their childhood (IBD: 32.3 %, HC: 26,5 %, χ^2^ (1) = 0.263, *p* = 0.608). Both groups did not differ in the number of traumatic events experienced in later life (IBD: M = 1.52, SD = 1.56; HC: M = 1.11, SD = 1.30), however, the IBD group exhibited a higher level of PTSD symptoms compared to HC *t*_44_ = 4.126, *p* < 0.001. See Table 2 for further details.

### Childhood Traumatization and Interoception

**Figure 4:**
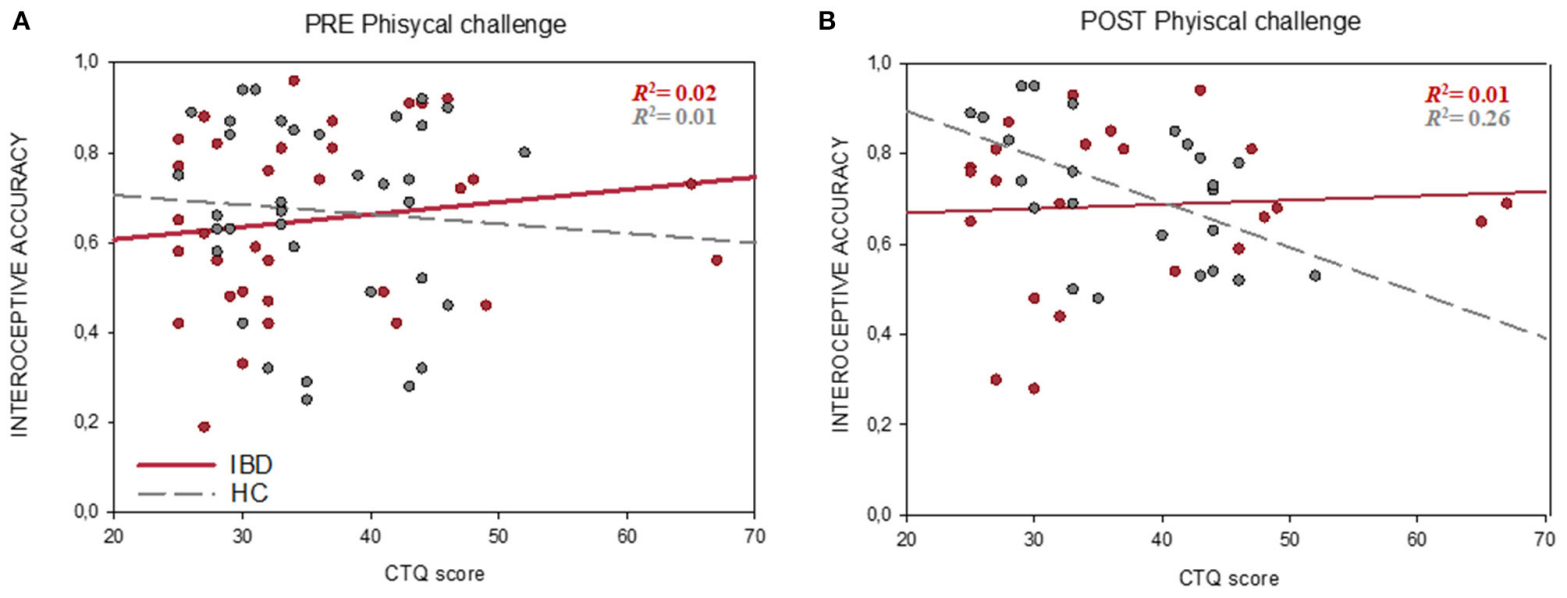
Associations between Interoceptive accuracy (HBT) and childhood traumatization (CTQ score) before **(A)** and after the physical challenge **(B)**. In contrast to the IBD group (red line), HCs (gray dashed line) showed a significant negative correlation between interoceptive accuracy and CTQ scores after the physical challenge. the physical challenge was implemented only in a subsample of 48 participants. CTQ, childhood trauma questionnaire; HC, healthy controls group; IBD, inflammatory bowel diseases group.

Regression analyses revealed no significant associations between interoceptive accuracy and CTQ in the present sample (all *p* > 0.05). However, exploratory analysis showed that interoceptive accuracy after the physical challenge was lower in those participants with higher CTQ scores in the HC group (*r*_24_ = −0.511, *p*_FDR_ = 0.022), but not in the IBD group (*r*_24_ = 0.070, *p*_FDR_ = 0.752, comparison between groups: *Z* = −2.06, *p* = 0.020) (Figure 4). CTQ scores did not show any significant links either to IS or interoceptive awareness (all *p* > 0.05).

### Childhood Traumatization and Emotional Processing

Linear regression analyses revealed no significant associations between CTQ scores and emotional processing (all *p* > 0.05).

### The role of ACE in Interoception, Emotional Processing and Their Association

**Figure 5:**
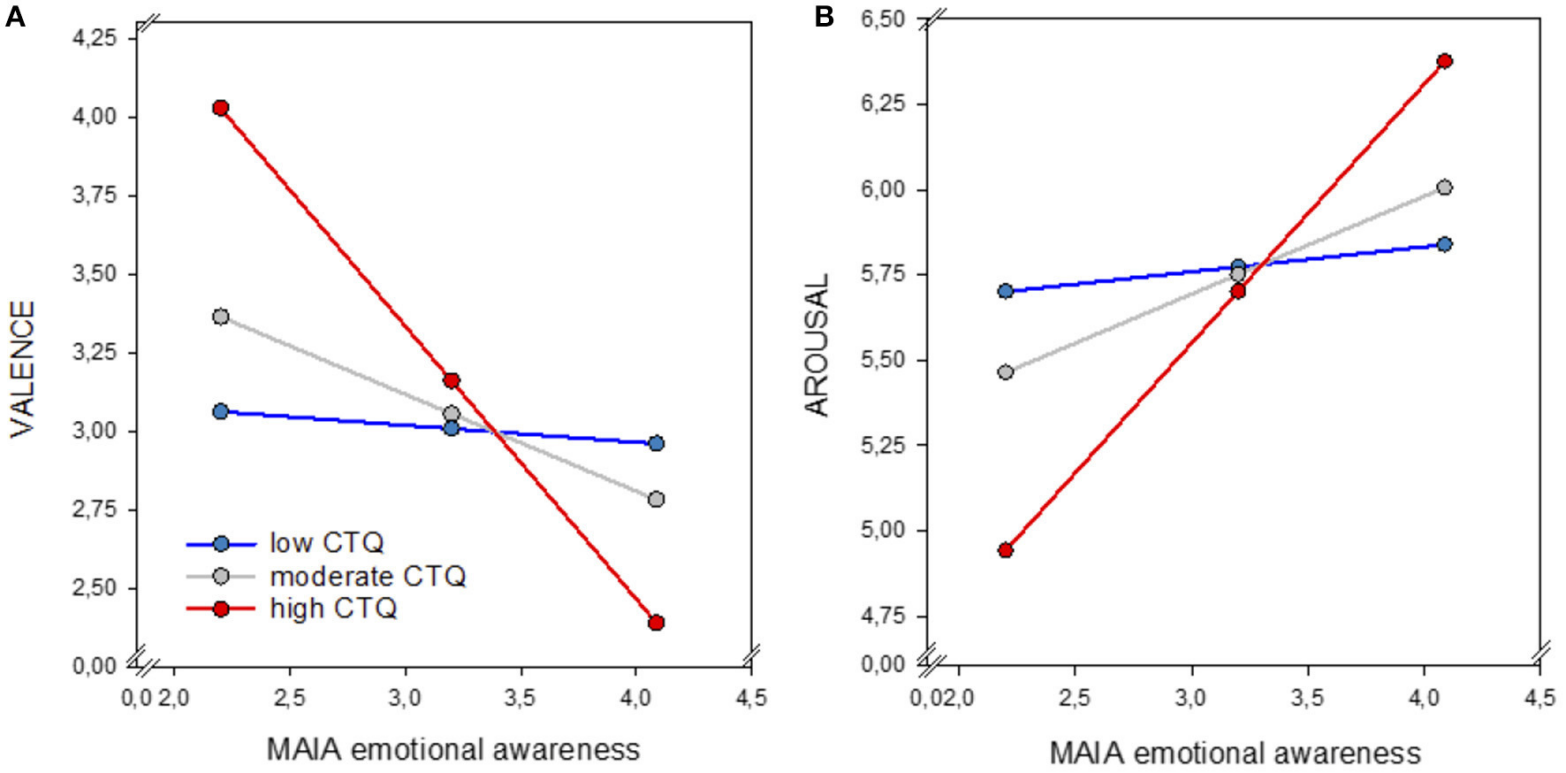
Moderation effect of childhood traumatization (CTQ) on the link between IS_emotional awareness_ (MAIA) and perceived valence (1 = very unpleasant, 8 = very pleasant) **(A)** and arousal (1 = low arousal, 8 = high arousal) **(B)** of negative stimuli. Participants reporting higher CTQ scores (red line) and high IS_emotional awareness_ reported higher negative valence and arousal compared to individuals exhibiting high IS_emotional awareness_ but moderate (gray line) or low (blue line) traumatization. IS, interoceptive sensibility; CTQ, childhood trauma questionnaire.

Moderation analyses showed no significant effects of CTQ scores on the link between interoceptive accuracy and emotional processing (all *p* > 0.05). However, CTQ severity significantly moderated the association between IS_emotional awareness_ and valence ratings of negative stimuli (*R*^2^= 0.22, *F*_3, 61_ = 5.588, *p* = 0.002). A significant interaction was found between IS_emotional awareness_ and CTQ scores (*b* = −0.05, *p* = 0.009), indicating that severer traumatization strengthens the negative relationship between IS_emotional awareness_ and valence intensity. Individuals with severe histories of ACE and higher IS rated negative stimuli as more negative compared to individuals with higher IS but no histories of childhood traumatization (Figure 5A).

For arousal ratings, analyses revealed a non-significant trend effect of CTQ on the association between IS_emotional awareness_ and perceived arousal of negative stimuli (*R*^2^ = 0.12, *F*_3, 61_ = 2.725, *p* = 0.052). A significant interaction between IS_emotional awareness_ and CTQ scores was found only for the highest CTQ severity (*b* = 0.76, *p* = 0.007) but not for the lower CTQ scores (*b* = 0.28, *p* = 0.09). These findings indicate that only severe ACE significantly amplified the positive relationship between IS_emotional awareness_ and arousal, suggesting that individuals reporting higher levels of childhood traumatization and high IS, do experience negative stimuli as more arousing compared to individuals with high IS but no or low levels of childhood maltreatment (Figure 5B).

### Heart Rate and Heart Rate Variability

#### Heartbeat Tracking Task

Mean HR during the HBT did not differ significantly between IBD and HC before the physical challenge (*t*_67_ = 1.490, *p* = 0.141). However, mean HR differed significantly before and after the physical challenge (main effect “time”: *F*_1, 46_ = 5.372, *p* = 0.025) and showed a non-significant trend for group differences (main effect “group”: *F*_1, 46_ = 3.284, *p* = 0.076; “time × “group”: *F*_1, 46_ = 0.059, *p* = 0.809). Thus, HR increased after the physical challenge in both groups, indicating that the implemented manipulation was successful.

Mean RMSSD during the HBT did not differ significantly between both groups (*t*_67_ = 0.367, *p* = 0.714). However, mean RMSSD differed significantly before and after the physical challenge (main effect “time: *F*_1, 46_ = 18.10, *p* < 0.001) and differed between both groups only depending on the presence of physical challenge (“time × “group”: *F*_1, 46_ = 9.66, *p* = 0.003; main effect “group”: *F*_1, 46_ = 0.24, *p* = 0.625;). Both groups exhibited increased HRV after the physical challenge. In the IBD group, however, this increase was less strong compared to HC.

#### Emotional Processing Task

Due to technical problems, baseline ECG data of three participants could not be analyzed. Mean HR and RMSSD at baseline and during the emotional processing task did not differ either in general or between both groups (all *p* > 0.05). For further details see Supplementary Table 1.

Childhood traumatization was not associated with alterations in cardiac activity in the whole sample (all *p* > 0.05). In IBD, higher CTQ scores were associated with higher HR at baseline (*r*_31_ = 0.420, *p* = 0.019). A non-significant trend association between mean RMSSD after the physical challenge and CTQ total score in the IBD group was found (*r*_23_ = −0.376, *p* = 0.077). No associations between cardiac activity and childhood traumatization could be found in HC. Because of the exploratory nature of these analyses, we did not correct for multiple comparisons (for more details see Supplementary Table 1.).

## Discussion

The present study investigated alterations of interoception and its role in emotion processing in IBD. This is the first study to assess interoceptive abilities as multidimensional construct implementing both behavioral and self-report methods. Therefore, we were able to investigate changes in different facets of interoception in IBD and to link these to emotion processing. Furthermore, associations between interoceptive abilities and emotion processing were investigated while taking childhood traumatization into account.

### Interoceptive Accuracy

Our findings indicated no differences between IBD and HC in the objective ability to track and perceive internal bodily sensations. These results are in line with previous studies using the same experimental paradigm in somatoform disorders (77), fibromyalgia (78) and irritable bowel syndrome (24). Contrary to out hypothesis, IBD patients did not exhibit greater interoceptive accuracy of cardiac signals. One possible mechanism explaining this finding could be provided by current theories of somatization suggesting that the perception of bodily sensations is based on previously formed symptom-related memory structures (79, 80). Potentially, IBD patients might be more focused on disease-specific visceral signals, which in turn captures cognitive resources and might impede an attentional shift to other internal signals such as their heartbeats. However, future studies need to assess interoceptive accuracy of other internal sensations (e.g., gastric signals) in IBD compared to healthy individuals, in order to provide empirical evidence whether disease-specific bodily sensations might be perceived more accurately. We implemented a short physical challenge to investigate whether activation of the cardiovascular system might influence individuals' abilities to track their heartbeats differentially in healthy individuals and patients with IBD. Our results showed that both groups improved as a non-significant trend in their interoceptive accuracy after the short physical exercise, indicating that induction of increased sympathetic activity elicits increased interoceptive accuracy. To our knowledge, this is the first study to investigate interoception by implementing a physical challenge in adults. Indeed, after the short physical activation participants improved in their interoceptive accuracy, showing that it might be useful to investigate interoceptive abilities after induced physiological arousal.

### Interoceptive Sensibility

Although both groups did not differ in their objective abilities to perceive visceral signals (heartbeats), our findings indicate that IBD patients do differ in their subjective interoceptive abilities regarding two main facets of IS: the tendency how to cope with unpleasant bodily sensations (IS_not-*distracting*_) and their ability to be focused on the body when experiencing various emotions (IS_emotional awareness_). Our results show that IBD patients exhibit a stronger tendency to use distraction as a coping mechanism when experiencing discomfort and physical pain. Coping strategies modulate the effects of psychological distress on illness experience in IBD (81). In line with our findings, previous research demonstrated that IBD patients often use self-distracting and avoidance strategies to cope with IBD symptoms (82). In general, distraction is the most common coping style for managing pain in chronic pain patients (83).

The current study expands existing findings on the association between emotional processing and interoception in IBD by showing a greater awareness of the connection between emotional states and bodily sensations in IBD patients. While the perception of visceral signals *per se* does not seem to be changed in remitted IBD patients, it is the appraisal of these sensations, which might be altered. When experiencing an emotion, IBD patients may appraise their bodily sensations differently from healthy individuals by putting greater emphasis on the perceived changes in the body, resulting in grater emotional awareness. Empirical findings from clinical and fundamental research have already demonstrated the physiological effects of gut microbiota on emotional processes, including emotion recognition. Tillisch et al. (84) could show a reduced midbrain connectivity activity and therefore altered activity in brain areas associated with vigilance to emotional stimuli in individuals after a 4-week therapy with probiotics (84). Alterations in gut microbiota, as frequently observed in IBD, might be linked to changes in emotion and sensory related brain regions (85), leading to changes in emotional awareness. Individuals with superior interoceptive abilities perceive emotions as more intense (86), which might be due to greater attention to emotion-related bodily changes and greater awareness of these mind-body associations. From a neurobiological perspective, the neural circuits involved in processing visceral information overlap with those involved in emotional processing (87, 88).

### Interoception and Emotional Processing

Our results indicated no group differences in the perceived valence of emotional stimuli. However, when evaluating their subjectively experienced arousal, IBD patients exhibited a greater arousal increase when being presented with neutral compared to positive stimuli. Interoceptive accuracy was not related to emotional processing in the present study. In contrast, IS_emotional awareness_ was associated with perceived valence and arousal of negative stimuli. Individuals exhibiting superior IS_emotional awareness_ experienced negative stimuli with a higher intensity, accompanied by higher levels of perceived arousal. However, these associations could be found only in the IBD group but not in HC (Figure 3). Our findings indicate that only emotional awareness as part of individual's interoceptive sensibility and not one's interoceptive accuracy influenced the perception of emotional stimuli. This pattern implies that altered processing of negative emotional content among IBD patients is not connected to changed perception of physiological stimuli but to their appraisal in the context of the emotional experience. These results could, therefore, indicate that the ability to identify and attribute physical sensations to certain emotional states (e.g., increased muscular tension and anger) might intensify patient's tendency to link bodily sensations to environmental triggers. In line with this interpretation, findings from fMRI studies also showed that individuals with high emotional awareness exhibit stronger emotion reactivity (89, 90). This might imply that IBD patients who are more aware of the physiological manifestations of emotions in the body may appraise these as more intense. Furthermore, neural correlates of emotional awareness include brain regions involved in the perception of both interoceptive signals and emotional cues (87, 91), suggesting that higher emotional awareness may be reflected by more efficient information exchange between these brain structures (92). It is conceivable that, although the perception of internal signals (heartbeats) did not seem to be changed, IBD patients might experience more differentiated bodily feelings during emotional experience as result of a changed neural functional connectivity (93). Since our results indicate that only the perception of negative stimuli was linked to emotional awareness, future studies need to investigate whether these effects can be explained by functional and structural changes in brain regions known to be relevant for the processing of negative emotions, visceral sensations, and interoceptive attention, such as the interior insula (91, 94).

### The Role of Childhood Traumatization

In contrast to previous findings, the IBD sample in the current study did not report higher levels of ACE compared to HC. A recent study found a significant percentage of IBD patients to have at least one ACE, stressing the possibility that childhood trauma might influence the course of IBD shown by a higher use of health care resources (37). However, in other population-based studies the link between maltreatment during childhood and IBD later in life was found only for Ulcerative colitis, but not for Crohn's disease (4). One possible explanation why the prevalence of ACE was not increased in our IBD sample might be that patients reporting current and/or lifetime psychiatric disorders were not included in the study. Early life stress has been repeatedly linked to higher risk for developing mental health problems. Previous studies investigating the prevalence ACE and IBD took primarily lifetime major depression and generalized anxiety disorders into account (4), but no further psychopathologies. However, none of these studies managed to disentangle the effects of ACE on IBD as well as on anxiety or depression symptoms by now. Further studies are required to investigate whether the interplay between interoception, emotion processing and ACE in IBD patients is influenced by psychiatric co-morbidities.

To the best of our knowledge, this is the first study to investigate the role of childhood maltreatment in interoception, emotional processing and their association. Our findings revealed no associations between ACE and interoceptive accuracy in IBD. Interestingly, we observed that in HC, ACE was negatively related to interoceptive accuracy only after the physical challenge. The implemented physical exercise was used to induce an increase in participants' heart rate and thereby enhanced cardiac signals perception (95). Our findings could, therefore, reflect the long-term negative impact of childhood maltreatment on individual's abilities to perceive visceral signals under conditions of physiological activation. Chronic early life stress leads to altered physiological response to arousal and dysregulation of the brain-body signaling (38, 39). In line, various trauma-related disorders (e.g., PTSD) have been repeatedly linked to altered physiological reactions to external threat cues (96, 97). In this case, threat perception might lead to maladaptive coping mechanism such as suppression and denial of bodily sensations due to overwhelming physiological reactions. In line with this interpretation, physical detachment is often observed in early-traumatized individuals as coping mechanism under high-stress conditions. Our results are also in line with Schaan et al. (38) who found a negative relationship between early life stress and interoceptive abilities only after an acute psychological stressor has been induced. Our findings suggest, however, that a physical stressor is also an eligible method to affect interoceptive accuracy and might be a useful approach to reveal possible associations between interoception and childhood traumatization.

Adverse childhood experiences have been repeatedly linked to impairments in affective processing (98). However, studies investigating the role of childhood traumatization on the link between interoception and emotion processing are sparse. We observed that the association between IS_emotional awareness_ and emotional processing of negative stimuli is moderated by severity of ACE independent of IBD diagnosis. This is a novel and interesting finding suggesting that among individuals with histories of childhood maltreatment, greater emotional awareness is associated with stronger intensity of negative affect and higher arousal to negative environmental stimuli. Previously, an attentional bias toward negative stimuli in individuals with ACE have been shown, indicating greater responsiveness to negative cues, heightened emotional response to possible threats and difficulties disengaging from negative emotional content (99). In the context of emotional experience, individuals with histories of childhood maltreatment might use their bodily signals in a more efficient way in order to track and recognize possible threats.

Finally, exploratory analyses of the data revealed higher PTSD symptoms severity including negative alterations in cognitions and mood, hyperarousal and reactivity in the IBD group. Some evidence indicates a higher risk for developing PTSD symptoms in IBD due to the challenges of the disease course most of the patients' experience (44). In summary, these findings emphasize the influence of early life adversity and higher frequency of posttraumatic stress symptoms in IBD. Since posttraumatic stress has been linked to worse IBD course through behavioral and physiological pathway, this might indicate a new possible target for intervention improvement (100).

## Limitations

Finally, some limitations of the present study have to be addressed. First, although we aimed to investigate a bigger sample (*N* = 120), the final sample consisted of 69 participants. Thus, given the smaller sample size larger effect size was needed to detect significant effects. *Post-hoc* sensitivity analyses showed that our study was sensitive to detect effect sized of *d* = 0.68 with 80% power in contrast to *d* = 0.52 for the planned sample size of *N* = 120). Moreover, it should be mentioned that although all IBD patients were in clinical remission, some of them reported minor health problems (e.g., knee pain), whereas all healthy control participants did not declare any health related complaints. In line with other studies using this task, we found relatively poor mean interoceptive accuracy scores between 0.50 and 0.70 (77, 101). Thus, the observed variance in both groups was quite low. It should be emphasized that there is a considerable body of evidence from physiological studies supporting the construct and criterion validity of the HBT (95, 102, 103). Thus, the reported poor validity of interoceptive accuracy measures in some studies result to a large extent from insufficiently controlled experimental environments or non-standardized changes in participant's physiological reactions (e.g., changing body posture during the task) (104). Previous studies found a strong association between cardiac and gastric sensitivity suggesting the presence of a general sensitivity for interoceptive cues across the cardiac and gastric modalities (47, 48). Following these considerations, we decided to use the HBT as a measure of the general interoceptive abilities in IBD. However, a recent study by Ferentzi et al. (105) has suggested that different interoceptive accuracy tasks reveal significant associations only when belonging to the same sensory modality, indicating HBT as a not specific measure of gastrointestinal interoceptive accuracy. In contrast, Whitehead and Drescher (106) could show a moderate correlation between heartbeat perception and perception of gastric contractions, when using the Heartbeat discrimination task, indicating a generalized tendency to be aware of visceral signals. Since both cardiac and gastric signals are transmitted to the brain partially through the vagus nerve, it is conceivable that their perception is closely related. Furthermore, one may critically discuss whether the correspondence between the observed interoceptive accuracy during the heartbeat tracking task and the self-reported interoceptive sensibility indeed reflect the construct of interoceptive awareness (14). A recent model proposed by Murphy et al. (107, 108) stressed the need for a careful differentiation between individual's interoceptive accuracy and interoceptive attention. Accordingly, interoceptive awareness of one's interoceptive accuracy as a metacognitive construct should be assessed as the relationship between one's behavioral performance and the corresponding awareness of particularly this performance, for example, measured by ratings of the participants' confidence in the accuracy of their performance. Thus, in the current study the approach to estimate interoceptive awareness corresponds more closely to the concept of interoceptive attention as to interoceptive awareness when conceptualized as a metacognitive construct [see (107)]. In consequence, further studies are required to investigate whether IBD patients' interoceptive awareness assessed as confidence ratings on their HBT performance might be affected. Finally, it should be mentioned that the severity of ACE in the present sample was only low to moderate (60), which might be due to the exclusion of individuals reporting current or lifetime psychiatric diagnoses. IBD patients reporting moderate traumatization, but no histories of mental disorders might represent a subgroup of patients with a less severe IBD course or resilient individuals who exhibited an adaptive coping with the experiences of traumatization. This restricts the generalizability of our findings to IBD patients with comorbid mental disorders. Since the significance of childhood traumatization in IBD might be underestimated in the current sample, our findings on the influence of ACE in IBD need to be interpreted with caution.

## Conclusions

In conclusion, IBD patients did not exhibit changes in the accuracy to perceive bodily signals such as their heartbeats. Our results demonstrate that the ability to attribute certain physiological sensations to emotional states intensifies the experience of negative emotions among IBD patients. As this population is quite prone to emotional distress and emotion dysregulation, future psychotherapeutic treatments should target patients' appraisals of physiological feedback during negative emotional states. Finally, the present findings point toward the important effect of early life stress on the interaction between mind and body, suggesting that individuals with histories of childhood traumatization might use their bodily sensations more efficiently in order to recognize negative emotional content and possible threats.

## Data Availability Statement

The raw data supporting the conclusions of this article will be made available by the authors, without undue reservation.

## Ethics Statement

The studies involving human participants were reviewed and approved by Ethics Committee of the Medical Faculty Mannheim, Heidelberg University. The patients/participants provided their written informed consent to participate in this study.

## Author Contributions

KA, WR, and SL designed the study, wrote the protocol, and wrote the first draft of the manuscript. KA, TL, and WR recruited the sample. KA performed data collection and conducted all statistical analyses. SL and WR provided substantive and conceptual feedback on all drafts. All authors contributed to and have approved the final manuscript.

## Conflict of Interest

The authors declare that the research was conducted in the absence of any commercial or financial relationships that could be construed as a potential conflict of interest.
