# Supplementary material for: Multidimensional Assessment of Interoceptive Abilities, Emotion Processing and the Role of Early Life Stress in Inflammatory Bowel Diseases
Source: Front Psychiatry. 2021 Jun 24;12:680878. doi: 10.3389/fpsyt.2021.680878 (PMC8264143; doi:10.3389/fpsyt.2021.680878)
Supplement: Supplementary file 1 [file Table_1.docx]

Supplementary Material

**Supplementary Material Table 1.**

**Heart rate and heart rate variability**

Repeated measures ANOVA with a between-subjects factor ‘group’ (IBD/HC) and repeated measures factor ‘time’ (baseline/time) revealed no differences in HR and HRV (RMSSD).

HR:
main effect ‘time’: *F*(1,64)=1.195, *p*=.278

main effect ‘group’: *F*(1,64)=0.416, *p*=.521

interaction effect ‘time’ x ‘group’: *F*(1,64)=0.079, *p*=.780

RMSSD:
main effect ‘time’: *F*(1,64)=0.191, *p*=.663

main effect ‘group’: *F*(1,64)=0.355, *p*=.554

interaction effect ‘time’ x ‘group’: *F*(1,64)=2.215, *p*=.142

Correlations between HR, RMSSD, and CTQ (Pearson’s correlation coefficients), p-values not corrected for multiple comparisons

|  | Whole sample | | IBD | | HC | |
| --- | --- | --- | --- | --- | --- | --- |
|  | *r* | *p* | *r* | *p* | *r* | *p* |
| HR baseline | .199 | .110 | .420 | .019 * | -.058 | .742 |
| HR Pre challenge | .038 | .761 | .151 | .401 | -.082 | .639 |
| HR Post challenge | .000 | .999 | .057 | .797 | -.073 | .734 |
| HR Emotion processing | .014 | .913 | .178 | .320 | -.158 | .366 |
| RMSSD baseline | -.071 | .574 | -.240 | .194 | .216 | .212 |
| RMSSD Pre challenge | -.070 | .573 | -.161 | .370 | .115 | .511 |
| RMSSD Post challenge | -.153 | .304 | -.376 | .077 (*) | .104 | .630 |
| RMSSD Emotion processing | -.021 | .863 | -.249 | .162 | .230 | .184 |
